# Enhanced methane steam reforming activity and electrochemical performance of Ni_0.9_Fe_0.1_-supported solid oxide fuel cells with infiltrated Ni-TiO_2_ particles

**DOI:** 10.1038/srep35981

**Published:** 2016-10-24

**Authors:** Kai Li, Lichao Jia, Xin Wang, Jian Pu, Bo Chi, Jian Li

**Affiliations:** 1 School of Materials Science and Engineering, Xi’an Shiyou University, Xi’an 710065, China; 2 Center for Fuel Cell Innovation, State Key Laboratory for Materials Processing and Die & Mould Technology, School of Materials Science and Engineering, Huazhong University of Science and Technology, Wuhan 430074, China

## Abstract

Ni_0.9_Fe_0.1_ alloy-supported solid oxide fuel cells with NiTiO_3_ (NTO) infiltrated into the cell support from 0 to 4 wt.% are prepared and investigated for CH_4_ steam reforming activity and electrochemical performance. The infiltrated NiTiO_3_ is reduced to TiO_2_-supported Ni particles in H_2_ at 650 °C. The reforming activity of the Ni_0.9_Fe_0.1_-support is increased by the presence of the TiO_2_-supported Ni particles; 3 wt.% is the optimal value of the added NTO, corresponding to the highest reforming activity, resistance to carbon deposition and electrochemical performance of the cell. Fueled wet CH_4_ at 100 mL min^−1^, the cell with 3 wt.% of NTO demonstrates a peak power density of 1.20 W cm^−2^ and a high limiting current density of 2.83 A cm^−2^ at 650 °C. It performs steadily for 96 h at 0.4 A cm^−2^ without the presence of deposited carbon in the Ni_0.9_Fe_0.1_-support and functional anode. Five polarization processes are identified by deconvoluting and data-fitting the electrochemical impedance spectra of the cells under the testing conditions; and the addition of TiO_2_-supported Ni particles into the Ni_0.9_Fe_0.1_-support reduces the polarization resistance of the processes ascribed to CH_4_ steam reforming and gas diffusion in the Ni_0.9_Fe_0.1_-support and functional anode.

On-cell methane (CH_4_) reforming in Ni-based anodes is an attractive option for directly using CH_4_-based fuels for solid oxide fuel cells (SOFCs) with high fuel efficiency and simplified system design^1^,^2^. CH_4_ steam reforming is a catalytic process for commercial production of H_2_ or syngas at a H_2_:CO molar ratio of 3:1 according to the endothermic reaction of


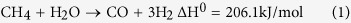


Excessive addition of H_2_O will further converts CO to CO_2_ by the slightly exothermic water gas shift (WGS) reaction^3^,^4^,^5^.


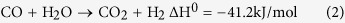


If these reactions are taking place in the anode of an SOFC, H_2_ is consumed via electrochemical oxidation to generate electrical power^6^,^7^, forming by-product of H_2_O. Such *in-situ* formed H_2_O is simultaneously used for CH_4_ steam reforming, which reduces the amount of externally added H_2_O to improve the electrical efficiency of the SOFC system.

However, for on-cell CH_4_ reforming in Ni-based anodes, coking is frequently observed in the anode when steam/carbon (H_2_O/CH_4_) ratio is low, since Ni catalyzes CH_4_ decomposition that produces deposited carbon in the form of filament or particle via either CH_4_ cracking or the Boudouard reactions as follow


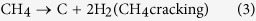



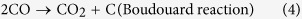


The soot-like carbon particles are distributed on the surface of Ni particles, occupying the active sites for electrochemical reaction and the pores for fuel gas transport^8^; and the carbon filaments formed by carbon diffusion into/precipitation out the Ni particles^9^ disintegrate the Ni-cermet anode by lifting out the Ni particles from the anode (dusting).

It has been demonstrated that infiltration of oxides, such as rare-earth doped CeO_2_^10^,^11^,^12^, BaO^13^ and CaO-MgO^14^, into the Ni-based anode is an effective way to enhance its coking resistance by suppressing carbon formation and promoting steam-carbon reactions. Although TiO_2_ has not been investigated in SOFCs, it was used as a support in catalysts for steam reforming of hydrocarbons (methanol^15^, ethanol^16^ and glycerol^17^), CO_2_ reforming of CH_4_^15^,^18^ and CO oxidation^19^; and high coking resistance was demonstrated in CH_4_^20^ and ethanol^16^ reforming. Stimulated by these investigations, TiO_2_ was evaluated in direct-CH_4_ SOFCs for the enhancement of CH_4_ on-cell reforming in the present study.

Compared with electrolyte- and electrode-supported SOFCs, metal-supported SOFCs have some advantages in the aspects of electrical/thermal conductivity and mechanical ductility; consequently, the temperature distribution in and tolerance to thermal cycle of the cell are improved^21^,^22^. In our previous study, Ni-Fe alloy-supported SOFCs were investigated with the purpose of using wet (3 vol.% H_2_O) CH_4_ as the fuel, and high performance (0.6 V at 0.4 A cm^−2^ and 650 °C for 50 h^7^) was achieved. However, the Ni_0.9_Fe_0.1_-support used was not fully resistant to carbon deposition, and carbon lumps were formed in its large pores. In order to develop metal-supported direct-hydrocarbon SOFCs, Ni_0.9_Fe_0.1_-supported SOFCs were prepared with NiTiO_3_ infiltrated into the Ni_0.9_Fe_0.1_-support. It was expected that NiTiO_3_ would be reduced into TiO_2_-supported Ni particles in H_2_ to enhance CH_4_ reforming activity and resistance to carbon deposition of the Ni_0.9_Fe_0.1_-supported cells.

## Results

### Materials and cell characterization

**Figure 1:**
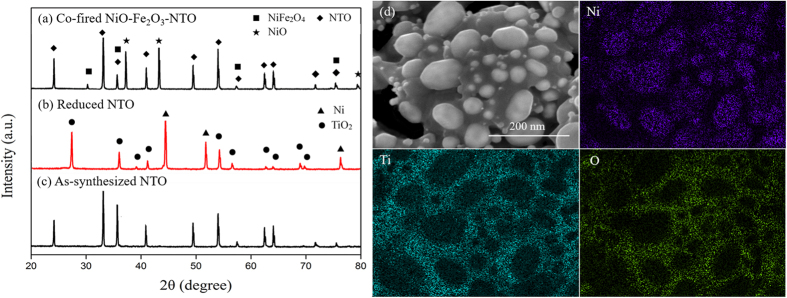
XRD patterns of (**a**) as-synthesized NTO powder (1000 °C for 2 h in air), (**b**) reduced NTO powder (650 °C for 2 h in H_2_), (**c**) co-fired NiO-Fe_2_O_3_-NTO powder mixture (1000 °C for 2 h in air) and (**d**) EDS mappings of Ni, Ti and O for TiO_2_-supported Ni particles.

Figure 1a–c show the XRD patterns of the as-synthesized and reduced NTO and co-fired powder mixture of NiO, Fe_2_O_3_ and NTO. The as-synthesized NTO demonstrated a perovskite structure of NiTiO_3_ (JCPDF# 76-0334), and the reduced product was a mixture of TiO_2_ (JCPDF# 21-1276) and Ni (JCPDF# 04-0850). Figure 1d shows EDS mappings of Ni, Ti and O for the mixture. It indicates that the bright granules in surface are identified by EDS as metallic Ni, and the dark areas rich in Ti and O. Based on this result, it is expected that the infiltrated NTO particles on the surface of the scaffold of the cell support be reduced into TiO_2_-supported Ni (0) particles. It was confirmed in our previous study^7^ that the sintered NiO-Fe_2_O_3_ cell support is consisted of two phases of NiO and NiFe_2_O_4_, and its reduced form is Ni_0.9_Fe_0.1_ alloy. With NTO powder added, the co-fired NiO-Fe_2_O_3_-NTO mixture contained NiO, NiFe_2_O_4_ and NTO (Fig. 1a), which indicates that NTO was chemically compatible with NiO and NiFe_2_O_4_ at temperatures up to 1000 °C and would remain as an independent phase in the scaffold of the sintered NiO-NiFe_2_O_4_ cell support.

**Figure 2:**
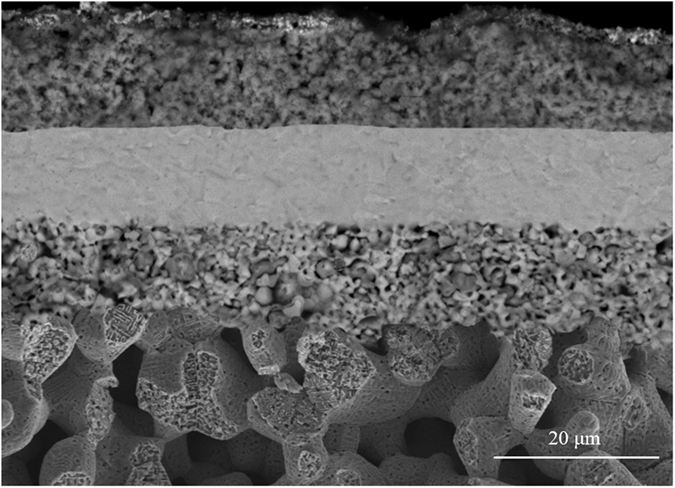
Fractured cross-sectional microstructure of a Ni_0.9_Fe_0.1_-support cell.

**Figure 3:**
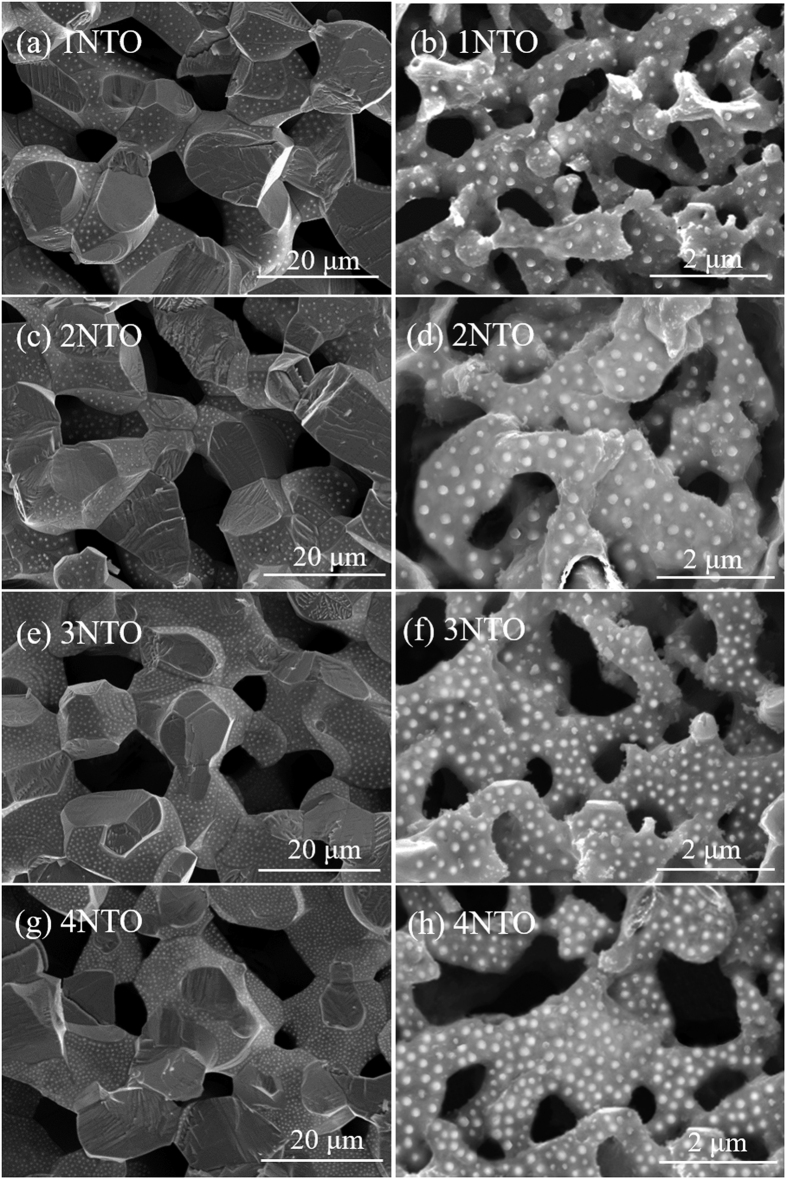
Fractured cross-sectional microstructure of (**a**) sintered and (**b**) reduced Ni_0.9_Fe_0.1_-supports with various amounts of infiltrated NTO.

Shown in Fig. 2 is the SEM microstructure of the fractured cross-section of the reduced cell with Ni_0.9_Fe_0.1_-support. As observed previously^7^, the sintered NiO-NiFe_2_O_4_ cell support was reduced into a porous scaffold (58%) with a bimodal pore distribution. The average size of the large pores was around 10 μm, which is beneficial for fuel gas transport in the support to the functional anode; and the small pores within the stem of the scaffold give a high specific surface area that is beneficial for CH_4_ reforming reaction. The Ni-GDC functional anode was approximately 1αm thick and intimately in contact with the fully dense GDC electrolyte (~10 μm) and the porous cell support (~1 mm). The thickness of the BSCF-LSM cathode was averagely 15 μm. Figure 3 respectively present the microstructure of the sintered and reduced cell supports with various amounts of infiltrated NTO from 1 to 4 wt.% of the weight of the half cell (NiO-Fe_2_O_3_ anode-support | NiO-GDC anode | GDC electrolyte).

### Reforming activity of infiltrated Ni_0.9_Fe_0.1_-supports

CH_4_ reforming in the Ni_0.9_Fe_0.1_-support is a chemical process that *in situ* produces H_2_, which is electrochemically oxidized on the functional Ni-GDC anode to generate electrical power with byproduct of steam via the reaction of


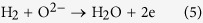


**Figure 4:**
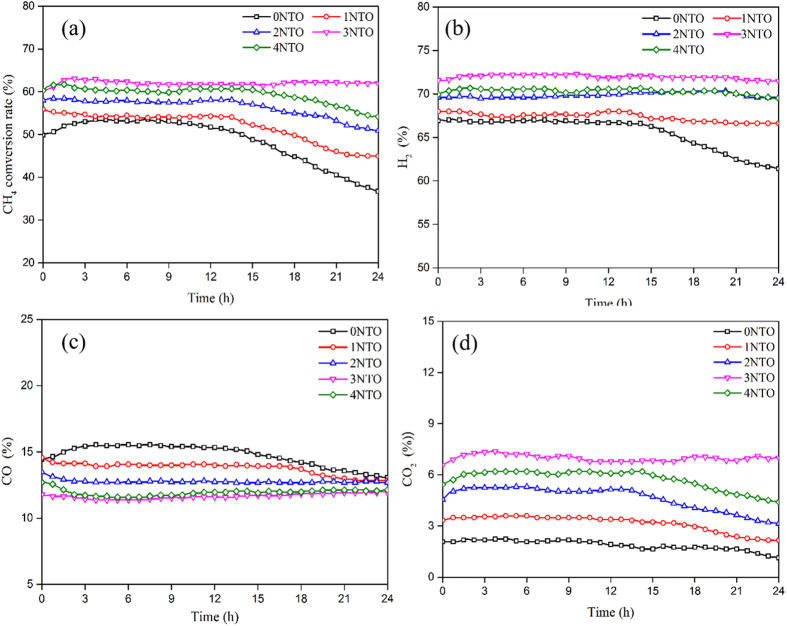
CH_4_ steam reforming of Ni_0.9_Fe_0.1_-supports with various amounts of infiltrated NTO at 650 °C and 1:1 CH_4_ to H_2_O ratio: (**a**) CH_4_ conversion rate and (**b**) H_2_, (**c**) CO and (**d**) CO_2_ concentrations in reformate.

Thus the reforming activity of the Ni_0.9_Fe_0.1_-support is of critical importance for the performance of the cell with on-cell CH_4_ reforming. Figure 4 shows the CH_4_ conversion rate and reforming product distribution at 650 °C in the Ni_0.9_Fe_0.1_-supports loaded with different amounts of TiO_2_-supported Ni particles. The initial values of CH_4_ conversion rate were approximately 50%, 55%, 58%, 61% and 60% for the Ni_0.9_Fe_0.1_-supports loaded with 0%, 1%, 2%, 3% and 4 wt.% of NTO (designated as 0NTO, 1NTO, 2NTO, 3NTO and 4NTO), respectively. This indicates that the addition of TiO_2_-suported Ni particles in the Ni_0.9_Fe_0.1_-support promoted its reforming activity with a limit of 3 wt.% NTO, more than which the conversion rate decreased, possibly due to the over-cover of the reforming active sites on the surface of the Ni_0.9_Fe_0.1_ scaffold by TiO_2_ and increased surface area of the small Ni particles for carbon deposition. The CH_4_ conversion rate of 0NTO, 1NTO, 2NTO and 4NTO decreased obviously with time after approximately 12 h, only which of 3NTO remained relatively stable during the testing period of 24 h. The main reforming products were H_2_, CO and CO_2_ (Fig. 4b–d), and their concentrations varied accordingly with the testing time.

### Cell performance

**Figure 5:**
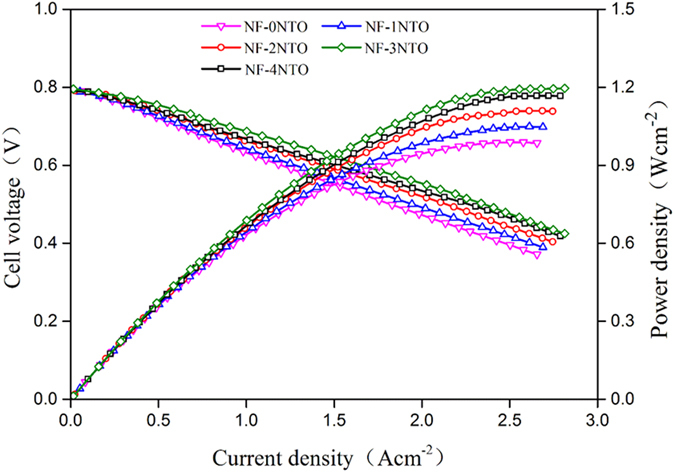
I-V-P curves of Ni_0.9_Fe_0.1_-supported cells with various amounts of infiltrated NTO in the Ni_0.9_Fe_0.1_-supports at 650 °C with wet CH_4_ as the fuel.

**Figure 6:**
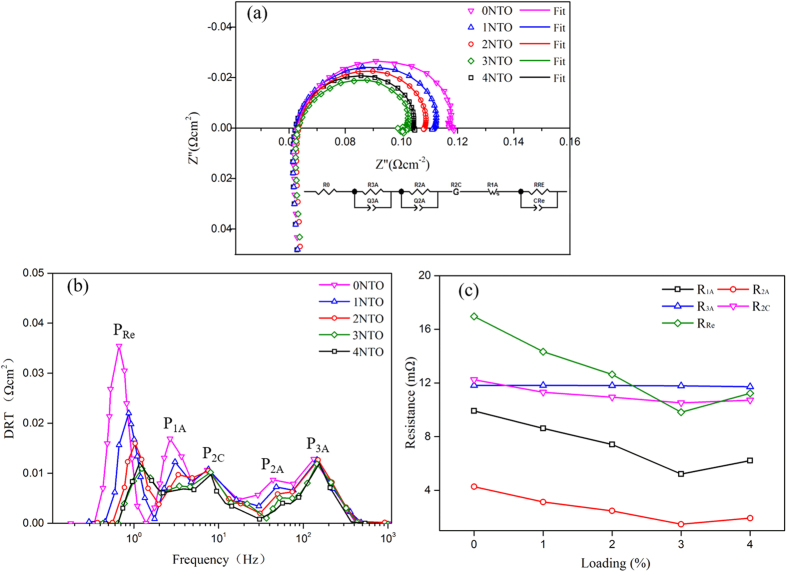
Impedance spectra at 650 °C and 0.4 A cm^−2^ (**a**), corresponding DRT (**b**) and polarization resistance of deconvoluted processes (**c**) of the Ni_0.9_Fe_0.1_-supported cells with various amounts of NTO in the Ni_0.9_Fe_0.1_-supports.

The cells with NTO-infiltrated Ni_0.9_Fe_0.1_-supports were evaluated at 650 °C with wet CH_4_ (3 vol.% H_2_O) as the fuel; Fig. 5 shows their initial I-V-P curves. The open circuit voltage (OCV) of all the cells was around 0.78 V, due to the partial electronic conduction of GDC electrolyte^23^. The maximum power densities increased from 0.99 to 1.20 W cm^−2^ as the NTO loading was increased from 0 to 3 wt.%. Further increasing NTO loading to 4 wt.%, it decreased to 1.17 W cm^−2^. Figure 6 shows the initial impedance spectra of the cells under a current density of 0.4 A cm^−2^ (Fig. 6a), from which the ohmic (*R*_O_) and polarization (*R*_P_) resistances were determined, and the corresponding distributions of relaxation time (DRT, Fig. 6b)^24^,^25^. The value of *R*_O_ of each cell was similar, around 0.063 Ω cm^−2^, and that of *R*_P_ varied in an opposite direction to the cell voltage and power density. This tendency of cell performance change with the amount of loaded NTO in the Ni_0.9_Fe_0.1_-support is consistent with that of the activity for CH_4_ steam reforming shown above, which suggests that cell performance improvement is due to the increased reforming activity of the Ni_0.9_Fe_0.1_-support and the consequent increase in the amount of H_2_ available for the anode reaction.

The DRT G(τ) was associated with the impedance Z(w) by the following expression:


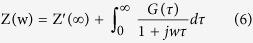


Where G(τ) is defined as the DRT of impedance Z, τ is relaxation time, Z′ (∞) is the limitation of the real part of Z as angular frequency w approaches infinity. Consequently, impedance could be represented as series connection of infinite number of parallel polarization resistor G(τ)dτ and a capacitor τ/G(τ)dτ. For a more detailed description of DRT method and application were referred^26^.

**Figure 7:**
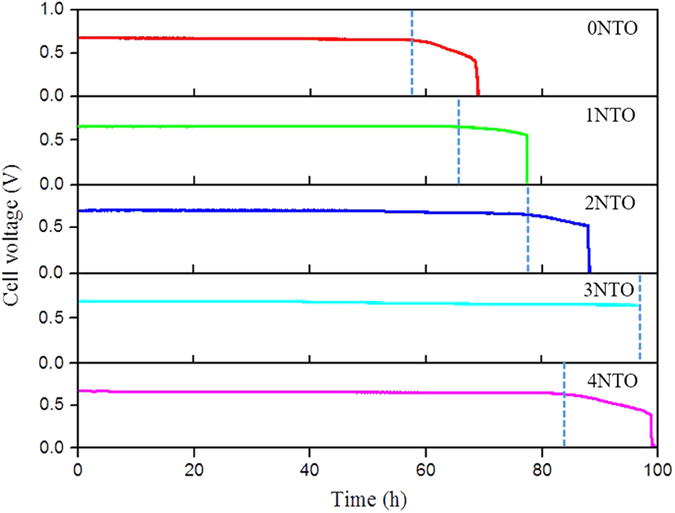
Cell voltage of wet CH_4_ fueled Ni_0.9_Fe_0.1_-supported cells with various amounts of NTO in the Ni_0.9_Fe_0.1_-supports as a function of testing time at 650 °C and a constant current density of 0.4 Acm^−2^.

**Figure 8:**
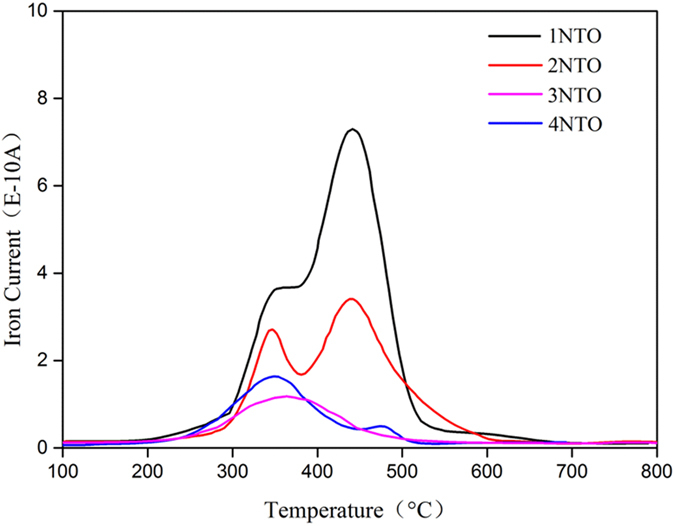
O_2_-TPO profiles of NTO infiltrated cells tested with wet CH_4_ as the fuel at 650 °C for up to 96 h.

After the initial evaluation, all the cells were further tested at 650 °C and a constant current density of 0.4 A cm^−2^ for up to 96 h; the results are shown in Fig. 7. The improvement on cell performance durability is in consistence with that on CH_4_ steam reforming activity. The cells with 0NTO, 1NTO, 2NTO and 4NTO Ni_0.9_Fe_0.1_-supports performed 67, 78, 90 and 96 h before the sudden drop of the cell voltage; and the cell with 3NTO Ni_0.9_Fe_0.1_-support outperformed the others, degrading linearly at a slow rate of 0.5 mV h^−1^ during the testing period. Post-test examination confirmed that the sudden voltage drop at the end of the test was caused by cell disintegration due to dusting of the Ni_0.9_Fe_0.1_-support. The linear voltage decrease, at nearly the same rate for all the cells, may represent the intrinsic cell degradation that needs further understanding for mechanism, whereas the non-linear voltage decrease is attributed to carbon deposition in the Ni_0.9_Fe_0.1_-support and functional anode. Since the deposited carbon remained in the cell, its amount can be quantified from the temperature-programmed oxidation (TPO) profile of the post-test cells, as shown in Fig. 8. The area of CO_2_ peak, an indication of the amount of CO_2_ formed from deposited carbon, were 7.89 × 10^−8^, 6.93 × 10^−8^, 2.61 × 10^−8^ and 3.15 × 10^−8^ for the cells with 1NTO, 2NTO, 3NTO and 4NTO Ni_0.9_Fe_0.1_-supports, respectively. These values support the explanation of the durability testing results and indicate that the cell with 3NTO anode-support is the most resistant to carbon deposition among the cells investigated.

## Discussion

According to previous studies^19^,^27^, the effectiveness of TiO_2_ on improving reforming activity can be attributed to its enhanced capability of H_2_O adsorption and consequently the coking resistance. It is the H_2_O adsorbed on the catalyst that increases the reforming activity^19^; and the prevalent presence of subsurface defects of TiO_2_ in reduced atmosphere, such as oxygen vacancies and Ti interstitials, enhances H_2_O adsorption due to surface relaxation and charge localization. On-cell methane reforming, constant adsorption of H_2_O in anode will shift the equilibrium reaction of Eqs (1) and (2) in a forward direction. Therefore, H_2_ and CO_2_ concentration increases whereas CO concentration decrease with increase in the amount of H_2_O. The increase in H_2_ concentration and the decrease in CO concentration subsequently prevent possible carbon formation by shifting Boudard reaction (Eq. 3) and decomposition of CH_4_ (Eq. 4) in a backward direction. In addition, the excess H_2_ reacts with oxygen ion from electrolyte to product electrical power and steam, which enhances the water-gas shift reaction and retards CH_4_ decomposition. In additional to the contribution of H_2_O adsorption on TiO_2_, the TiO_2_-supported Ni particles on the surface of Ni_0.9_Fe_0.1_ scaffold are also considered to increase the reforming activity, due to its known tendency to form a strong metal-support interaction (SMSI) between TiO_2_ support and Ni metal and widely used catalyst of CH_4_ and ethanol steam reforming^16^,^28^.

Based on the DRT shown in Fig. 6b and the results reported in a previous investigation^25^, five polarization processes were identified for individual cells, which are two high-frequency processes ascribed to the gas diffusion and charge transfer/ionic transport within the functional anode (P_2A_ and P_3A_), one high-frequency process associated with oxygen surface exchange and bulk diffusion within the BSCF-LSM cathode (P_2C_), one low-frequency process related to mass transport in the Ni_0.9_Fe_0.1_-support (P_1A_) and one low-frequency process attributed to CH_4_ reforming in the Ni_0.9_Fe_0.1_-support (P_Ref_). The contribution of each process to the total polarization resistance was obtained by data fitting the impedance spectra (Fig. 6a) using the complex nonlinear least-squares method and an equivalent circuit (inset in Fig. 6a) consisting of an ohmic resistor *R*_O_, two RQ elements for P_2A_ and P_3A_, a Gerischer element (G) for P_2C_, a generalized finite length Warburg element (W) for P_1A_ and another RQ element for P_R_. The change of the polarization resistance for each process, R_1A_, R_2A_, R_3A_, R_2C_ and R_Ref_, with the amount of loaded NTO is demonstrated in Fig. 6c. R_3A_ and R_2C_ remained almost unaffected by NTO infiltration, since the cathode was identical for all the cells, and the electrochemical reaction in the functional Ni-GDC anodes was the same reaction of H_2_ oxidation^25^ regardless of the amount of NTO loaded in the Ni_0.9_Fe_0.1_-support. The resistance of diffusion of reformate in the Ni_0.9_Fe_0.1_-support and Ni-GDC functional anode, R_1A_ and R_2A_, decreased with increasing NTO amount till 3 wt.% and then increased at 4 wt.%, which reflects the amount change of H_2_ in the reformate. It is expected that higher concentration of H_2_ in the reformate lead to lower diffusion resistance in porous cell support and functional anode due to the high diffusivity of H_2_. R_Ref_ is assigned to CH_4_ steam reforming process; its change with the amount of loaded NTO in the Ni_0.9_Fe_0.1_-support is consistent with that of the reforming activity. According to the data-fitting results and discussions, it may be concluded that the cell performance improvement with NTO infiltration in the Ni_0.9_Fe_0.1_-support is attributed to the improved CH_4_ reforming activity and the decreased potential of carbon deposition; consequently the polarization resistances related to CH_4_ reforming and reformate transport processes are decreased.

NTO infiltration into Ni_0.9_Fe_0.1_-supports was investigated with the purpose of enhancing CH_4_ steam reforming activity, carbon deposition resistance and cell performance. Based on the obtained results and discussion, the following conclusions are drawn.

1. The activity of the Ni_0.9_Fe_0.1_-support for CH_4_ steam reforming is enhanced by infiltrated NTO, which is reduced into TiO_2_-supported Ni (0) particles in H_2_. The TiO_2_ improves the resistance to carbon deposition by adsorbing H_2_O, while the supported small Ni particles promote CH_4_ decomposition.
2. 3 wt.% of the weight of the half cell (anode-support | functional anode | electrolyte) is the optimal value for the amount of NTO infiltrated into the Ni_0.9_Fe_0.1_-support. Increased CH_4_ reforming activity lead to the improvement of cell performance, durability and resistance to carbon deposition.
3. The overall cell polarization resistance is contributed by five polarization processes associated with CH_4_ reforming (P_ref_), mass transport in anode-support (P_1A_), gas diffusion in functional anode (P_2A_), charge transfer within functional anode (P_3A_), and oxygen surface exchange and bulk diffusion within cathode (P_2C_). The addition of NTO into the Ni_0.9_Fe_0.1_-support reduces the polarization resistance of P_ref_, P_1A_ and P_2A_.

## Methods

### Cell fabrication

Ni_0.9_Fe_0.1_-supported cells were fabricated by tape casting-screen printing-sintering process. NiO (Haite Advanced Materials) and Fe_2_O_3_ (Sinopharm) powders were mixed at a Ni:Fe molar ratio of 9:1 and ball-milled for 24 h in xylene/ethanol solvent with fish oil (Richard E. Mistler, Inc.) as the dispersant, corn starch as the pore former, poly vinyl butyral (Solutia Inc.) as the binder and butyl benzyl phthalate and poly alkylene glycol (Solutia Inc.) as the plasticizer. The prepared slurry was cast into a tape with a dry thickness of ~1.2 mm, which was then die-cut into discs (25 mm in diameter) as the cell support, on which NiO (Inco)-GDC (10 mol.% Gd-doped CeO_2_, NIMTE, CAS) functional anode and GDC electrolyte were screen printed in sequence, followed by sintering at 1450 °C in air for 5 h. La_0.8_Sr_0.2_MnO_3_-coated Ba_0.5_Sr_0.5_Co_0.8_Fe_0.2_O_3_ (LSM-BSCF) cathode^29^ was then screen-printed on the sintered GDC electrolyte and sintered in air at 1050 °C for 2 h.

To introduce TiO_2_-supported Ni particles onto the stem of NiO-Fe_2_O_3_ scaffold (~40% porosity^30^), an aqueous solution containing Ti and Ni ions at the stoichiometric concentration of NiTiO_3_ (NTO) was prepared as follow. Tetrabutyl titanate (C_16_H_36_O_4_Ti, Sinopharm) was dissolved in a dilute nitric acid aqueous solution under stirring, and then stoichiometric amount of Ni nitrate (Ni(NO_3_)_2_·6H_2_O, Sinopharm) was added prior to the addition of citric acid (CA) and ethylenediamine tetraacetic acid (EDTA) as the chelants. The molar ratio of metal ions:CA:EDTA in the solution was 1:1:1.5. Ammonia solution was used to adjust the pH value of the solution to approximately 7. Such prepared solution was infiltrated into the pores of the sintered NiO-Fe_2_O_3_ scaffold and calcined in air at 1000 °C for 2 h to form crystallized NTO nano particles. This infiltration process was repeated to achieve the desired amounts of loaded NTO in the scaffold. The crystal structure of NTO and its chemical reactivity with NiO and Fe_2_O_3_ were determined by X-ray diffraction (XRD, X’Pert) using a NiO-Fe_2_O_3_-NTO powder mixture co-fired in air at 1000 °C for 2 h. The NTO powder was obtained by calcining the dried solution in air at 1000 °C for 2 h, and its reduced form (650 °C in H_2_ for 2 h) was characterized by XRD for phase identification and examined by using a scanning electron microscope (SEM, FEI sirion 200).

### Steam reforming activity evaluation

To evaluate the catalytic activity of the infiltrated Ni_0.9_Fe_0.1_-support for CH_4_ steam reforming, the NiO-Fe_2_O_3_ support sintered at 1450 °C in air for 5 h was sealed in a ceramic housing using a Ceramabond^TM^ sealant (Aremco Product, Inc.) and reduced at 650 °C in H_2_ for 2 h. Then a mixture of 10% CH_4_, 10% H_2_O and 80% He was fed into the porous support at a constant rate of 100 ml min^−1^. The steam content in the mixture was controlled by flowing dry CH_4_ and He gases through a saturator containing distilled water at 50 °C according to the following equation^31^.


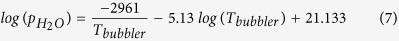


Compositional analysis of the effluent gas from the reactor was conducted with an on-line Pfeiffer Vacuum Mass Spectrometer. The steam reforming was performed at temperatures between 500 and 700 °C, and the CH_4_ conversion rate (X (%)) was estimated using the following equation.


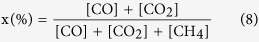


### Cell testing and characterization

The cell performance was evaluated at 650 °C with wet (3 mol.% H_2_O) CH_4_ as the fuel and ambient air as the oxidant at a flow rate of 100 ml min^−1^. Using a power supply of Solartron 1480A in 4-probe mode, the current density (i)–voltage (V)-power density (P) polarization curves were obtained at a scanning rate of 5 mVs^−1^ from 0 to 1 V, and electrochemical impedance spectra (EIS) were acquired within a frequency range from 100 KHz to 0.01 Hz and an AC signal amplitude of 10 mV. The microstructure of the cell was examined by using a SEM. The resistance to carbon deposition of (the amount of deposited carbon in) the Ni_0.9_Fe_0.1_-supported cell was characterized by temperature-programmed-oxidation (TPO) method at a flow rate of 20 ml min^−1^ of pure oxygen.

## Additional Information

**How to cite this article**: Li, K. *et al.* Enhanced methane steam reforming activity and electrochemical performance of Ni_0.9_Fe_0.1_-supported solid oxide fuel cells with infiltrated Ni-TiO_2_ particles. *Sci. Rep.* **6**, 35981; doi: 10.1038/srep35981 (2016).
